# Planning for the future of the American Society for Microbiology’s Health Unit by the Council on Microbial Sciences

**DOI:** 10.1128/msphere.00633-25

**Published:** 2026-03-16

**Authors:** Austin J. Fox, Andrea M. Prinzi, Nancy W. Wamburu, Nicholas M. Moore, Michael M. Lieberman, Kileen Shier, Nancy L. Haigwood

**Affiliations:** 1 Wisconsin State Laboratory of Hygiene 37360 , Madison, Wisconsin, USA; 2 US Medical Affairs, bioMérieux, Salt Lake City, Utah, USA; 3 American Society for Microbiology 11003, Washington, DC, USA; 4 Division of Clinical Microbiology, College of Health Sciences, Rush University, Chicago, Illinois, USA; 5 University of Hawaii, John A Burns School of Medicine 50677, Honolulu, Hawaii, USA; 6 Quest Diagnostics 828715 , Chantilly, Virginia, USA; 7 Oregon National Primate Research Center, Oregon Health & Science University 88960, Beaverton, Oregon, USA; Virginia-Maryland College of Veterinary Medicine, Blacksburg, Virginia, USA

**Keywords:** vaccines, surveillance studies, diagnostics, veterinary microbiology, clinical microbiology, data integration, infectious disease, public health, outreach, clinical therapeutics

## Abstract

In February and March 2025, the American Society for Microbiology (ASM)’s Council on Microbial Sciences (COMS) hosted a series of virtual retreats to integrate the communities of Clinical Infections and Vaccines (CIV) and Clinical and Public Health Microbiology (CPHM) under the umbrella of the new scientific unit ASM Health. Representatives from these two communities invited experts and ASM members to reflect on the current state of the science, identify challenges hindering scientific advancements, and propose recommendations for overcoming these challenges. Sessions focused on progress in (i) vaccines, therapeutics, diagnostics, and global pathogen surveillance; (ii) improving data integration and cross-agency sharing; (iii) supporting the microbial health workforce; and (iv) gaining public support and confidence in microbiology. Four major recommendations emerged for ASM Health. First, increased support for microbiological science is crucial to ensure the advancement of vaccines, therapeutics, diagnostics, and global pathogen surveillance to mitigate infectious disease threats. Second, enhancement of data integration and sharing in real-time via information systems will facilitate a deeper understanding of disease epidemiology. Third, modern approaches to recruitment, career path, and profession awareness, and dynamic training programs are necessary to achieve microbial workforce balance. Fourth, microbiologists will benefit from outreach training and resources designed to restore public trust in an environment that questions science and evidence-based approaches. ASM is uniquely positioned to take a pivotal leadership role to develop these concepts into specific programs, as well as to enhance partnerships across the spectrum to innovate in funding, both for scientific research and public health.

## INTRODUCTION

In recent decades, the importance of microbiology to human and animal health has been in the spotlight with the emergence and spread of pathogens worldwide. From food safety to disease emergence and countermeasures, microbes and microbiology are frequently at the center of both scientific and, more recently, political discourse. Emerging infectious diseases are no longer rare anomalies—they have become defining challenges of our era. The rapid appearance of novel pathogens, alongside the reemergence of diseases once considered under control, underscores the fragility of our global health systems. With outbreaks such as SARS-CoV-2, mpox, avian influenza, drug-resistant tuberculosis, and the resurgence of measles and pertussis, we are witnessing a convergence of scientific, ecological, and societal forces that demand new approaches to microbial science and disease prevention. At the same time, diagnostic tools and biomedical technologies are evolving at an unprecedented speed, and we now generate and have access to more biological and clinical data than ever before. Yet, translating these advances into timely, coordinated public health responses in an environment of diminished scientific support and vaccine hesitancy remains a persistent challenge (1, 2).

The pace of diagnostic, vaccine, and therapeutic development during the COVID-19 pandemic demonstrated what is possible when political will, resources, regulatory flexibility, and scientific innovation align. Diagnostic tests, monoclonal antibodies, and vaccines were developed, validated, and deployed in record time (3), hand in hand with public health measures that were both disruptive and effective. Modeling has shown that non-pharmaceutical interventions that were enacted both reduced SARS-CoV-2 infections and substantially slowed the spread (4, 5). This approach saved lives and reshaped the expectations for how quickly we can respond to future threats. However, that same urgency revealed critical gaps in preparedness, data interoperability, laboratory capacity, healthcare delivery, and public trust. The paradox of having powerful tools and incomplete systems has become clear. Although we can sequence genomes in hours and produce candidate vaccines in weeks, building the infrastructure needed to support equitable deployment, provide clear communication, and organize sustained surveillance requires more time. Simultaneously, environmental change and human encroachment into previously undisturbed habitats are altering the dynamics of pathogen emergence (6). Wildlife, livestock, and human populations are coming into closer contact, thereby increasing the likelihood of zoonotic spillover events (7). These shifting patterns further complicate the surveillance landscape, making a unified, interdisciplinary response across sectors more essential than ever (8).

It is against this backdrop that the American Society for Microbiology (ASM) convened a retreat to reflect on the current state of clinical and public health microbiology and to chart a course for its future. ASM, founded in 1899, is one of the oldest scientific organizations dedicated to promoting microbial science globally. With more than 32,000 members, including scientists and healthcare practitioners, ASM advances microbial science through, among other things: conferences, journals and publishing, certifications, educational opportunities, career development activities, and advocacy. ASM enhances laboratory capacity worldwide by providing training and resources, creating a network for professionals in academia, industry, government, clinical, and public health settings, while seeking to foster a deeper understanding of microbial sciences among diverse audiences. The goals of the retreat were to (i) synthesize the current landscape of ASM Health-related science, including key advances and persistent barriers; (ii) identify strategic priorities and emerging opportunities that align with ASM’s mission; and (iii) better integrate clinical and public health microbiology across the broader microbial sciences. In doing so, ASM aims to foster collaboration, align scientific progress with public health needs, and define a roadmap for action that centers on science, resilience, and innovation.

ASM brought together 13 members of the Council on Microbial Sciences (COMS) with interests in clinical infections and vaccines (CIV) and clinical and public health microbiology (CPHM) in 2024. This Retreat Planning Committee identified four major priorities and designed sessions around these areas, with opening presentations from invited expert speakers or panelists, followed by participant discussion and synthesis. Sessions were held virtually over 4 days in February and March of 2025. Participants were from across the United States (Hawaii to New York) and all career stages, with a majority being mid- and senior-level professionals. They included clinical and public health microbiologists/lab directors/associate directors; assistant/associate/full professors; industry professionals, including field medical affairs representatives, senior scientists, and company founders/CEOs; research microbiologists; sociologists; educators/instructors; and ASM staff from various departments. ASM staff members, as well as ASM and COMS leaders, attended all sessions. ASM staff contributed to logistics, were panelists, took notes during breakout sessions, and facilitated discussions. Committee member and speaker affiliations are provided at the end of the article. Each retreat session is summarized in the sections below, followed by an overall set of recommendations endorsed by the COMS Retreat Planning Committee at the 2025 ASM Microbe 2025 Conference.

## ACCELERATING THE DEVELOPMENT OF VACCINES, THERAPEUTICS, DIAGNOSTICS FOR INFECTIOUS DISEASES

Nancy Haigwood, Michael Lieberman, Nicholas Moore, Andrea Prinzi, and Anthony Tran were the organizers for “Accelerating the Development of Vaccines, Therapeutics, Diagnostics for Infectious Diseases.”

### Vaccines

Mark Slifka, Ph.D., spoke on vaccines.

**Fig 1:**
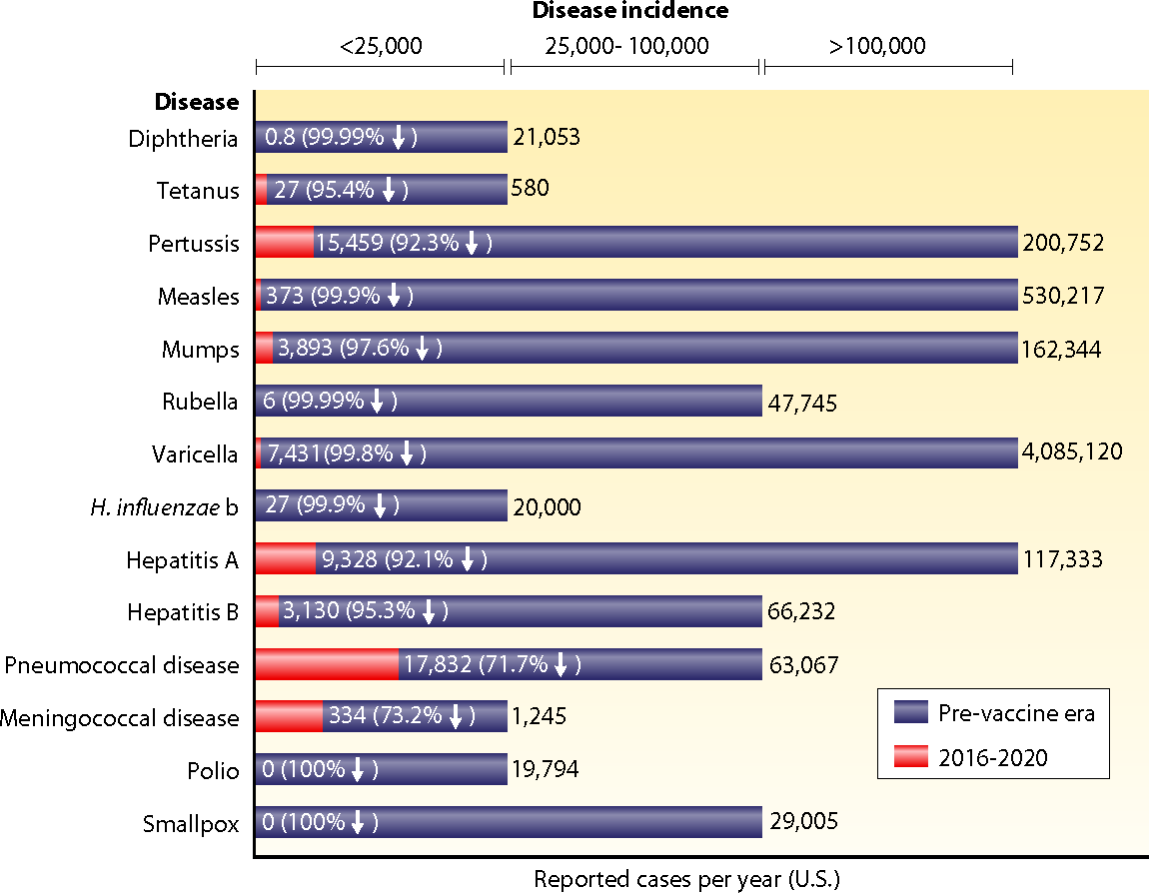
Childhood vaccines protect children and reduce the burden of disease (10), reprinted with permission. Fourteen major childhood diseases are listed on the left side of the graph, and the incidence of reported cases per year in the United States is shown as individual bars, with a scale at the top. In red is the incidence in 2016–2020, after vaccines were introduced, and in purple is the incidence in the pre-vaccine era. Note the absence of cases of polio and smallpox. The percentage reduction is denoted in the purple bar in parentheses with the downward arrow, next to the number of cases.

Legacy vaccines have played a crucial role in the success of global public health, significantly reducing mortality from diseases that once resulted in widespread fatalities (9). In the United States, the current risk of contracting tetanus is less than 1 in 10 million, and the risk for diphtheria is less than 1 in 1 billion, both of which are far lower than the 1 in 1.2 million risk of being struck by lightning. Vaccines for numerous other childhood infectious diseases have greatly reduced the incidence of these diseases compared to the pre-vaccine era (10) (Fig. 1). These remarkable outcomes have led to a reevaluation of booster recommendations. Recent evidence suggests that adults who received full childhood immunization against tetanus and diphtheria may not need additional boosters (11). However, the evolving field of vaccinology indicates that long-term vaccine effectiveness relies on more than just initial antibody levels. While advanced adjuvants (12) can enhance early immune responses (13), their impact on sustaining immunity and antibody longevity remains limited (14). This distinction is particularly important when designing vaccines for emerging infectious threats, where long-lasting protection may depend on a deeper understanding of immune mechanisms beyond the structure of antigens alone (15).

Vaccination against seasonal influenza has been the primary method of prevention of infection for many decades and is currently recommended for all persons aged 6 months or older who do not have contraindications (https://www.cdc.gov/flu/vaccines/vaccinations.html). Numerous studies have shown that vaccinated patients have reduced risk of hospitalization and death, and even in a year when the vaccine was mismatched to the circulating virus, it reduced children’s risk of severe life-threatening pneumonia by 75% (16). Widespread uptake of these vaccines over many years has saved millions of lives and has also allowed exploration of the complexities of vaccine effectiveness (17). Despite a 13-fold increase in the distribution of the influenza vaccine over the past 40 years, flu-associated mortality has not improved proportionally. Studies have shown that repeated annual influenza vaccinations may paradoxically decrease vaccine effectiveness. For instance, during the 2014–2015 season in Canada, viral genetic changes, combined with repeated vaccination, contributed to historically low vaccine effectiveness (18). Similarly, evidence from Japan indicates that individuals who were vaccinated in the prior season had significantly lower protection after vaccination in the following year compared to those who were unvaccinated in the prior season and then vaccinated. This effect was not seen in individuals with prior natural infections (19). Additionally, the widespread use of split-virus vaccines, which contain fragmented viral components, may limit immune responses to new viral strains (20), especially in immunologically naïve populations. In contrast, whole-virus vaccines tend to induce more durable immunity.

These challenges underscore the urgent need for long-term immunological studies to clarify the mechanisms driving vaccine durability (i.e., longevity of protective immunity) and immune modulation, driving increased efficacy in future vaccines. This understanding is vital for tailoring vaccine strategies and public health policies to effectively protect vulnerable populations. Moving forward, it is essential to develop next-generation adjuvants that not only enhance initial immune responses but also sustain long-term immunity, particularly for diseases such as tuberculosis, malaria, and HIV, which disproportionately impact low- and middle-income countries (LMICs). Vaccine development must also consider regional epidemiological and delivery challenges, including overcoming vaccine hesitancy through culturally sensitive communication and community engagement (1). Preparedness for emerging diseases requires robust infrastructure to support rapid vaccine development, the stockpiling of monoclonal antibodies as adjunct therapies, and other medical countermeasures to mitigate and reduce the spread of infectious disease outbreaks (2). Effective responses will depend on sustained collaboration across public and private sectors to optimize funding, resource allocation, and technology transfer (21).

### Therapeutics

Nancy L. Haigwood, Ph.D., spoke on therapeutics.

Alongside public health measures, vaccines, and diagnostics, therapeutics are a crucial part of infectious disease management and preparedness and have significantly reduced deaths due to infectious diseases (https://www.cdc.gov/mmwr/preview/mmwrhtml/mm4829a1.htm). Despite the spectacular success of antibiotics, the widespread use of antimicrobial agents in both the healthcare and food industries has led to increasing antimicrobial resistance (AMR) worldwide (22). Furthermore, effective drugs have not yet been discovered to treat some of the world’s major killers, and outbreaks of both novel and known pathogens require time and resources to devote to drug discovery. The timeline for drug discovery, refinement, development, and approval is not only long but also very expensive (23). Nonetheless, several deadly diseases for which there are no vaccines can now be managed with highly effective drug treatment (e.g., HIV and lifelong therapy) or even cured (e.g., hepatitis C and tuberculosis). Although the COVID vaccines are very effective in preventing severe disease, antiviral medications, such as nirmatrelvir and ritonavir (Paxlovid), are still needed to diminish the severity of SARS-CoV-2 infection in unvaccinated individuals.

Antibody therapeutics (IgG or plasma/serum) have been an important tool to limit disease outbreaks (e.g., diphtheria) for more than a century (24). The use of convalescent plasma (CP) or serum containing pathogen antibodies remains a powerful therapy to blunt infectious diseases; CP was used in over 0.5 million patients during the COVID-19 pandemic, saving many lives (25). However, there is inherent variability in individual immune responses to infection, meaning that the CP can be difficult to qualify and to reproduce on a large scale. More recently, such immunotherapies have been refined using high-affinity monoclonal antibodies (mAbs) directed to specific pathogen antigens and that are highly effective in blocking infection. Since their FDA approval in 1986, mAbs have provided passive immunity by direct delivery of pathogen-specific antibodies to both adults and infants (26, 27), offering immediate protection lasting from 1 to 6 months, in contrast to vaccines, which take time to trigger the body’s immune response. mAbs are particularly valuable for populations that cannot be vaccinated or in outbreak situations where rapid immunity is needed, such as in the case of recent Ebola outbreaks (28). In the United States, human mAbs are the standard of care for respiratory syncytial virus infections in newborns (https://www.cdc.gov/mmwr/volumes/72/wr/mm7234a4.htm). Additionally, combinations of HIV-specific mAbs have been successfully used to prevent HIV infection (29), as well as to suppress virus replication in established infections, allowing patients a reprieve from drug treatments. Although there has been significant progress in identifying viral-targeted antibodies, less attention has been given to discovering mAbs that target bacterial toxins or conserved metabolic pathways. This could open up new possibilities for the treatment of AMR pathogens. One strategy is to prepare for emerging diseases by stockpiling already discovered mAbs that are directed to the most lethal pathogens and that are the most likely to emerge (30).

Disadvantages of passive antibody therapies include escape from mAbs directed to quickly mutating viruses, such as SARS-CoV-2, the need for high doses and repeated administration to maintain protective levels in the blood and tissues, in addition to the need for the cold chain and clinic-based delivery. Some newer approaches include novel delivery of mAbs for respiratory infections; aerosolized mAbs can significantly reduce SARS-CoV-2 viral load and lung inflammation (31) in mice and in nonhuman primates. There is also the potential for *in vivo* antibody expression over the long term, even years, by vector-mediated gene delivery to muscle cells, which has shown effectiveness and safety in nonhuman primates (32), but significant safety questions remain to move this into the clinic. Nonetheless, challenges remain, such as high production costs, intellectual property issues, and limited global infrastructure. In the U.S., these obstacles are particularly pronounced for diseases that are considered rare or non-endemic. To move the therapeutics field forward to complement vaccine development, it is essential to invest more and foster sustained collaboration between the public and private sectors to strengthen discovery, funding, and manufacturing capacities, and to ensure equitable access to innovative therapeutics (33).

### Clinical diagnostics

Elizabeth Marlowe, MS, Ph.D., D(ABMM), spoke on clinical diagnostics.

The effective development and deployment of clinical diagnostics across national laboratory systems is a complex challenge that requires coordinated efforts from both public and private sectors. Laboratory capacity, resource availability, and focus vary widely in clinical and public health settings, each with unique needs and constraints. This diversity, combined with rapidly evolving scientific advances, emerging health threats, and limited funding, means that public health and clinical laboratories alone cannot meet the growing demand for timely, high-quality testing. Instead, integrated networks that include clinical laboratories, academic institutions, and public health programs must work together seamlessly to ensure equitable access to diagnostic services.

Central to this collaboration is the partnership between public health infrastructure and clinical and industry stakeholders, which proved vital for real-time evaluation of diagnostic performance and vaccine development during the COVID-19 pandemic (21). These partnerships also enable the development, evaluation, validation, and deployment of new diagnostic assays (34). However, private industry collaboration or support may be limited when the return on investment is uncertain. This gap highlights the need for public sector leadership and funding to support early-stage innovation and incentivize collaboration. Additionally, early and consistent engagement with regulatory bodies is crucial to establish clear and flexible validation protocols that can adapt to the evolving regulatory landscape, especially concerning the development and clinical use of laboratory-developed tests.

Achieving harmonization in diagnostic practices requires a strategic focus on standards rather than rigid standardization, which can hinder innovation and responsiveness. Diagnostic systems must remain agile and capable of rapid deployment on existing platforms while comprehensive validation continues. Local laboratory validation plays a critical role as an interim measure, provided that standardized materials and quality assurance tools are readily accessible. Efforts to harmonize essential components, such as defining what constitutes a high-quality clinical specimen (i.e., optimizing collection practices in the pre-analytical phase) and establishing equivalency across nucleic acid extraction platforms, are crucial to building resilience and addressing challenges identified in previous public health responses.

Underlying these scientific and operational challenges is the persistent issue of funding and value alignment in the diagnostic development pipeline. Diseases with limited commercial appeal, such as tuberculosis in high-income or low-prevalence settings, struggle to attract private investment, making public-private partnerships indispensable to meeting public health needs. Adopting investment frameworks based on successful vaccine development initiatives, including pre-allocated emergency funding, could accelerate diagnostic readiness. Moreover, ongoing collaboration with developers regarding platform selection, validation, and deployment is vital to ensure that new assays align with laboratory capabilities and clinical requirements, ultimately enhancing the nation’s capacity to respond to current and future health threats.

### Surveillance of global public health

Edwin Kamau, MS, Ph.D., spoke on surveillance of global public health.

Diagnostics serve as the foundation of global public health by enabling timely pathogen detection, surveillance, and responses, which are key components of effective epidemic containment and health security (35). Programs such as the World Health Organization’s International Pathogen Surveillance Network (IPSN) utilize pathogen genomics to enhance the detection and characterization of infectious agents globally, supporting data-driven public health decisions, such as vaccination strategies and resource allocation. The IPSN’s structure, which includes Communities of Practice, Country Scale-Up Accelerators, and donor coordination, illustrates the increasing emphasis on genomic surveillance as a vital tool for understanding transmission patterns and guiding outbreak responses.

Similarly, the U.S. Department of Defense’s Global Emerging Infections Surveillance program leverages established international partnerships and laboratory capacity to provide rapid biosurveillance information essential for protecting military and global populations (36). Other initiatives, such as the Pathogen genomic Diversity Network Africa (https://pathogens-dna.org/;) and the ASM’s laboratory capacity-building programs (https://asm.org:443/global-health/building-laboratory-capacity), concentrate on enhancing regional capacity and translating genomic data into actionable knowledge, especially in LMICs. Historical outbreaks further demonstrate the critical role of diagnostics in shaping effective global responses. The 2002–2003 SARS epidemic (37) highlighted the importance of rapid detection and transparent information sharing, coordinated by the WHO and international partners, which enabled containment within months. The 2014–2016 West African Ebola outbreak (38) emphasized the necessity for robust surveillance systems and international collaboration to manage widespread transmission in regions with limited infrastructure. More recently, genomic sequencing played a pivotal role during the COVID-19 pandemic (39) by identifying viral variants, informing vaccine updates, and guiding public health interventions worldwide. Additionally, the 2024 Marburg virus disease outbreak in Rwanda illustrated how integrating diagnostics with high-quality clinical care and coordinated trials can significantly improve outcomes, even in resource-constrained settings (40–42). This underscores diagnostics’ potential not only to detect but also to shape comprehensive epidemic control strategies.

Although the value of these diagnostic approaches has been well-established, challenges persist. LMICs continue to face significant barriers in building and sustaining effective laboratory and surveillance infrastructure. These challenges include limited laboratory capacity and data infrastructure, chronic underfunding, frequent dependency on short-term grants, and a shortage of skilled personnel and training opportunities (43). Political instability and logistical obstacles further complicate efforts to strengthen health systems.

A major factor influencing these systems in developing countries is the dependence on foreign funding, which brings both opportunities and risks. On the positive side, external funding can provide critical resources, technical expertise, and capacity-building opportunities that may otherwise be unavailable. However, it can also lead to misaligned priorities where donor interests overshadow local needs and foster dependency, making it difficult for LMICs to maintain operations independently over the long term. To transition toward more sustainable and self-sufficient systems, LMICs must focus on investing in laboratory infrastructure, workforce development, and accessible local diagnostic testing. Diversifying funding sources and strengthening political engagement and advocacy are essential to reduce reliance on external donors. Establishing a balanced partnership with international funders requires clearly articulating national priorities, ensuring strong local ownership of initiatives, and collaboratively planning to align programs with both local needs and long-term sustainability goals.

Looking ahead, advancing diagnostics demands continued investment in infrastructure, workforce training, and sustainable funding models, particularly in LMICs, where challenges such as limited lab capacity, political hurdles, and reliance on foreign aid persist. Diversifying funding sources and promoting local ownership and collaboration are critical in building resilient surveillance systems that address immediate needs while ensuring long-term sustainability. Emerging tools like genomic sequencing, wastewater surveillance, and rapid point-of-care diagnostics provide promising opportunities for earlier detection, population-level monitoring, and decentralized testing—all vital for efficient outbreak response. As an African proverb states, “Until the lion has his or her own storyteller, the hunter will always have the best part of the story.” This emphasizes the need for local leadership and narratives in shaping the future of global diagnostics and health security.

## IMPROVING AND INTEGRATING DATA ACCESS AND SHARING ACROSS DISCIPLINES AND AGENCIES

Indira Kudva, Nicholas Moore, and Andrea Prinzi were the organizers for “Improving and Integrating Data Access and Sharing Across Disciplines and Agencies.”

### Veterinary medicine and the One Health perspective

Kelli Maddock, DrPH, MS, MLS(ASCP)^CM^M, spoke on veterinary medicine and the One Health perspective.

Data sharing and surveillance of AMR in veterinary medicine face unique challenges that complicate a cohesive One Health approach. In the One Health perspective, animals, ecosystems, and humans are integral and equal pieces of a unified and healthy environment. Although humans and animals are closely connected, AMR surveillance systems, diagnostic standards, and treatment practices remain largely siloed, with key differences in clinical breakpoints, pharmacokinetics/pharmacodynamics, and regulatory restrictions across species. Veterinary care often occurs in decentralized or rural settings without direct links to pharmacies or laboratories. The risks of AMR spread from food animals—via meat products, waste runoff, or contaminated crops are significant (44). Despite existing veterinary antimicrobial susceptibility testing (AST) standards (e.g., from the Clinical Laboratory Standards Institute [CLSI]; https://clsi.org/shop/standards/vet01/), interpretation is complex, and resistance observed in animals does not always correlate with resistance risk in humans. The absence of species-specific clinical breakpoints for AMR testing forces reliance on human-derived standards, which may not reflect appropriate therapeutic responses in animals and can undermine both animal health and food safety. Data sharing for food animals is further hindered by concerns about economic impact and food safety perceptions (45), potentially leading to underreporting of antibiotic use. AMR surveillance infrastructure is supported by networks like USDA’s National Animal Health Laboratory Network (https://www.aphis.usda.gov/labs/nahln) and FDA’s Veterinary Laboratory Investigation and Response Network (https://www.fda.gov/animal-veterinary/science-research/veterinary-laboratory-investigation-and-response-network). However, these efforts also focus on foreign animal disease outbreaks and pet food safety. Companion animal data sharing is less contentious, but standardization issues persist, including overreporting of antibiotic-target organism combinations without cascade reporting (first-line options and broad-spectrum activity to narrow-spectrum, targeted antimicrobials), which presents a common stewardship gap. Active education is improving reporting practices, but efforts are needed to update definitions of multidrug resistance across species and promote harmonized, responsible data sharing aligned with One Health principles.

## IMPROVING DATA SHARING AND INTEROPERABILITY

Panelists for “Improving Data Sharing and Interoperability” were Raj Dash, MD, Ph.D.; Riki Merrick, MPH; Angie Maxted, MS, DVM, Ph.D., DACVPM; and Kelli Maddock, DrPH, MS, MLS(ASCP)^CM^M.

Data sharing and interoperability remain persistent challenges in clinical microbiology and laboratory medicine, especially within the One Health framework that seeks to integrate human, veterinary, and environmental health systems. Interoperability defines a model in which all computer systems and the software within an information technology workspace function within the same, easily accessible, and translatable language format. Although the COVID-19 pandemic spurred significant advancements in data infrastructure for human health, these gains have not been evenly distributed across sectors. Fragmented implementation of established standards (https://www.nlm.nih.gov/oet/ed/healthdatastandards/02-200.html), such as Logical Observation Identifiers Names and Codes (LOINC) for test identification (https://loinc.org/get-started/what-loinc-is/), Systematized Nomenclature of Medicine—Clinical Terms for coding (https://www.nlm.nih.gov/healthit/snomedct/index.html), and Health Level Seven Fast Healthcare Interoperability Resources for data exchange (https://ecqi.healthit.gov/fhir/about), limits real-time information sharing between public health laboratories, clinical systems, and surveillance platforms. When data sharing between clinical and public health laboratories works, it is often unidirectional, commonly favoring clinical data over public health and not the reciprocal due to many reasons, including local/state regulations, privacy concerns, and data sharing capabilities. Although human clinical data increasingly flows into electronic health records, enabling improved patient care and outbreak detection, similar interoperability lags in veterinary and environmental health systems, creating critical vulnerabilities in our global surveillance capacity.

This fragmentation is not due to a lack of technical standards, but rather inconsistent adoption, insufficient funding, and unclear regulatory mandates. Effective surveillance, especially for threats like AMR, requires terminology, definitions, and data structures that are universally interpretable or easily translatable across disciplines. Yet many veterinary laboratories still lack access to laboratory information systems (LIS) that support structured, interoperable data exchange. These disparities reflect broader structural imbalances: while human health systems benefit from targeted investments and infrastructure, veterinary and environmental sectors remain under-resourced and less integrated into national and global health data ecosystems.

To address these challenges, initiatives like the Systemic Harmonization and Interoperability Enhancement for Laboratory Data framework (https://www.fda.gov/medical-devices/diagnostic-data-program/systemic-harmonization-and-interoperability-enhancement-laboratory-data-shield) and the Laboratory Interoperability Data Repository are working to standardize how diagnostic metadata are cataloged and exchanged. These efforts aim to reduce regulatory burden, enhance outbreak detection, and streamline communication between clinical and public health systems. Industry collaborations, such as those led by the In-Vitro Diagnostic (IVD) Industry Connectivity Consortium (https://ivdconnectivity.org/), are promoting machine-readable mappings and harmonized vocabularies to automate data flows. Likewise, the CLSI has emphasized the importance of semantic interoperability, ensuring a consistent understanding of laboratory data through guidance provided by AUTO17 software (https://clsi.org/shop/standards/auto17/), which encourages manufacturers to align test results with structured formats such as LOINC to Vendor IVD Test Results Mapping (LIVD; https://ivdconnectivity.org/livd-specification/).

Still, real-world adoption of these practices remains variable. Even when technical standards exist, there is no universally accepted data model that encompasses human, animal, and environmental health. Differences in how systems define specimens, collection methods, or AST breakpoints make integration complex. Moreover, implementation efforts are often hindered by limited resources, unclear guidance, and a lack of incentives, particularly in veterinary and environmental domains, which are frequently overlooked in national data modernization strategies.

Moving forward, a more unified and strategic approach is essential. Regulatory bodies must provide clear, enforceable guidelines that prioritize interoperability, while public and private sectors collaborate to develop scalable infrastructure. Financial incentives and targeted funding should support under-resourced sectors in adopting modern LIS platforms and training a skilled workforce. Equally important is the development of integrated databases that merge human, animal, and environmental health data to enable cross-sector analysis and timely responses to emerging threats. Efforts to harmonize data collection and reporting standards, particularly for AMR (22), will be key to improving data comparability and public health outcomes.

## ATTRACTING, EDUCATING, AND RETAINING THE MICROBIAL HEALTH WORKFORCE OF THE FUTURE

Austin Fox, Kileen Shier, and Nicholas Moore were the organizers for “Attracting, Educating, and Retaining the Microbial Health Workforce of the Future.” The speakers were Susan M. Harrington, Ph.D. D(ABMM) MLS(ASCP)^CM^, and Joshua Kropp, MLS(ASCP)^CM^.

The clinical and public health microbiology workforce is facing a pivotal moment (46), shaped by pandemic-driven disruptions, funding challenges resulting in declining opportunities in public health and basic research, a surge in retirements attributed to the aging of the baby boomer generation, and shifting expectations among new professionals within the United States. According to recent data, over 80% of laboratories in the United States report at least one open position (47, 48), with vacancy rates highest among bench-level medical laboratory professionals. The American Society for Clinical Pathology (ASCP) regularly conducts laboratory vacancy surveys. The most recent survey, based on data gathered in 2024, suggests that clinical microbiology labs have staff (e.g., non-supervisor) vacancy rates of 8.5% and that 14% of microbiology employees are expected to retire in the next 5 years. In 2024, there was a notable decline among respondents in microbiology laboratories that report being required to hire certified individuals into technical roles (52.8% in 2204 vs 64.0% in 2022). While the authors of the ASCP vacancy survey note that current vacancy rates are lower than in 2022 for many specialty areas, they are still higher than vacancy rates in the pre-COVID-19 era (49). At the same time, the field is projected to grow steadily, with a 5% increase expected over the next decade (https://www.bls.gov/ooh/healthcare/clinical-laboratory-technologists-and-technicians.htm), translating to 344,200 jobs and 24,200 annual openings. Despite this growth, the loss of experienced personnel due to funding cuts, retirement, burnout, and career switching, especially at the bench and supervisory level, is creating significant knowledge gaps, straining onboarding and training processes, and weakening mentorship pipelines that are vital for skill development and retention.

The influx of bachelor’s-trained microbiologists without formal medical laboratory science (MLS) certification offers a partial solution to staffing shortages, but it places a considerable burden on laboratories to provide on-the-job training, often without dedicated funding or standardized protocols. These costs are rarely tracked but are substantial, particularly for public health and hospital labs operating under tight budgets. This challenge emphasizes the need for clear workforce data, including standardized job titles and metrics on the proportion of non-certified staff, to better align educational resources and institutional investments. Furthermore, in states that require licensure of clinical laboratory testing personnel, non-certified laboratorians cannot be used in this capacity.

Educational models must also evolve to meet the complexity of today’s microbial health landscape. Traditional MLS and medical laboratory technologist programs remain essential, but they often lack specialized training in molecular diagnostics, automation, and applied microbiology. In contrast, innovative hybrid models, e.g., the ASM–Weber State University Microbiology Certificate Program (https://asm.org:443/certifications/asm-weber-state-university-microbiology-certificat), provide flexible, affordable pathways to MLS certification through online coursework and clinical rotations at local laboratories. This program addresses national shortages by training bachelor’s-level scientists for certification under the ASCP Board of Certification Route 3 (https://apps.ascp.org/services/boc/BOC_Cert_RouteRequirement) and currently operates in over 26 cities, serving as a scalable blueprint for workforce expansion.

**Fig 2:**
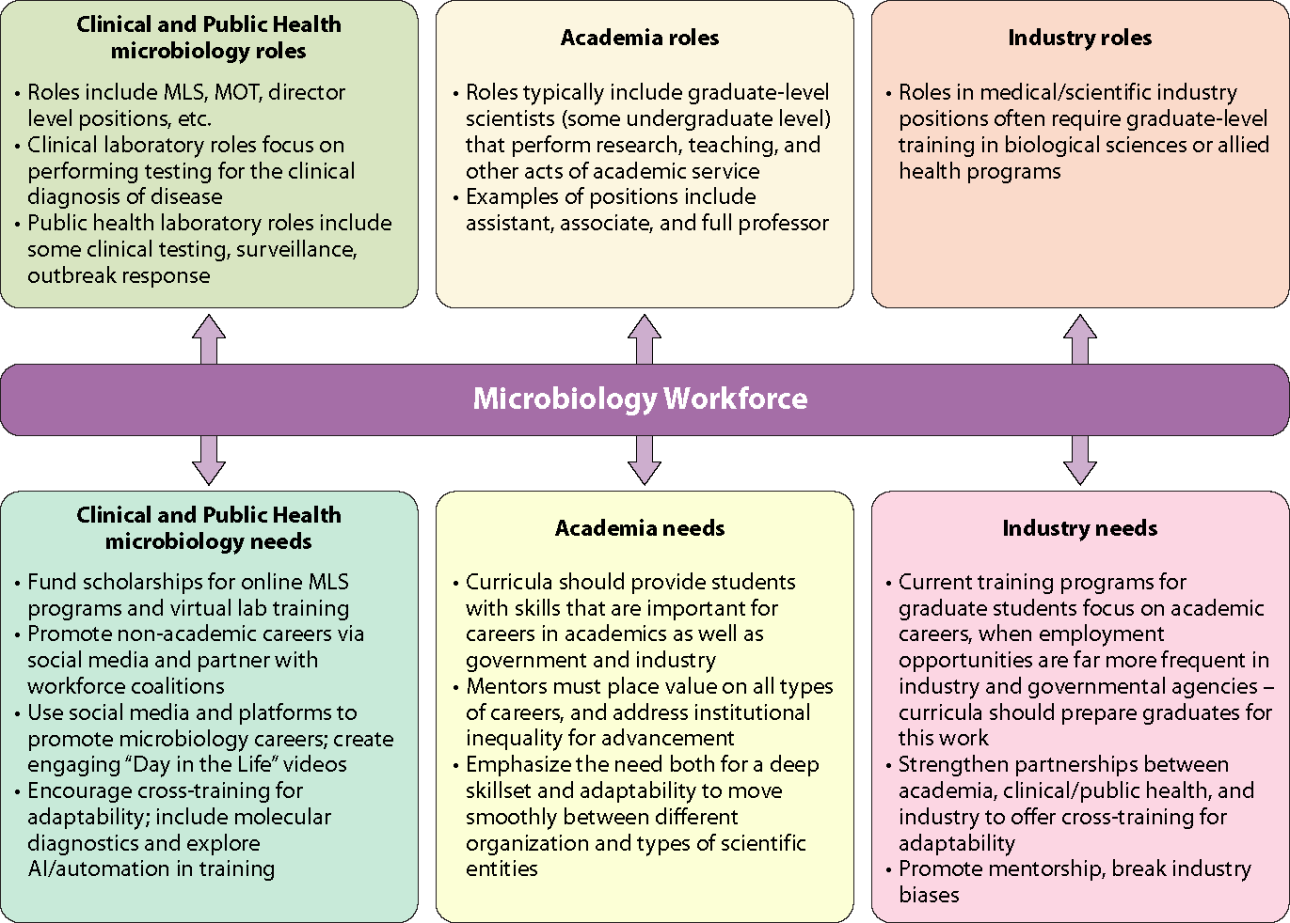
Current roles of clinical and public health, academia, and industry in educating and attracting the microbiology workforce are listed in the upper boxes. The lower boxes indicate some of the more compelling needs in these three sectors in order to build capacity and expertise for the future.

Improving recruitment and retention also requires a cultural shift in how microbiology careers are presented and supported. Many students discover laboratory careers informally, often too late to pursue aligned educational paths. Strategic outreach through social media, online platforms, and high school counselor engagement is critical. College professors at the undergraduate biology level should provide practical information on medical laboratory career paths. Tools such as the ASCP career roadmap (https://www.ascp.org/careers-fellowships/careers-in-the-laboratory) have proven effective in raising awareness, but broader efforts are needed to promote early and informed career exploration (Fig. 2). Moreover, retention strategies should emphasize inclusive leadership, mentorship, and professional development. Integrating business, leadership, and artificial intelligence (AI) literacy into training can help laboratorians navigate cross-sector career paths across clinical, public health, academic, and industry roles (50). By modernizing education, increasing career visibility (51), and supporting adaptive training environments, we can build a resilient, well-prepared microbial health workforce equipped for both the current demands and future threats.

## GAINING PUBLIC SUPPORT AND CONFIDENCE IN MICROBIOLOGY AND PUBLIC HEALTH TO ENHANCE HEALTH

The organizers for “Gaining Public Support and Confidence in Microbiology and Public Health to Enhance Health” were Kileen Shier and Tara Smith. The panelists were Jacinda Abdul-Mutakabbir, PharmD, MPH; J. R. Kane, BA; and Jennifer A. Reich, MA, Ph.D.

Public comprehension of microbiology’s influence on health and daily life remains limited and inconsistent. Even among healthcare professionals, there are gaps in understanding how microbiological related phenomena, such as avian influenza outbreaks, can translate to real-world consequences, such as disruptions in food supply chains. These gaps often stem from the way biology is taught: abstracted from lived experience, overly technical, and difficult to translate into everyday relevance. Many people have deeply personal, historical experiences with science, ranging from the legacy of unethical clinical trials to intergenerational skepticism, which shape how they receive public health messaging (52). To build trust, science communication must begin with empathy, acknowledging these lived experiences and the reality that individuals are not inherently anti-science but often disconnected from how scientific conclusions are reached (53).

The COVID-19 pandemic highlighted deep limitations in science communication infrastructure (54). Public health communication has often been reactive, inconsistent, and overly centralized. Scientists are typically trained in technical rigor, not public engagement, which can lead to information gatekeeping or messages that alienate rather than inform. Moreover, the rise of personalized medicine to derive individual risk assessments has inadvertently fostered a misunderstanding of population-level science. For example, messaging that COVID-19 poses higher risks to older adults was interpreted by younger individuals as a reason to forego vaccination, revealing a critical failure to explain statistical nuance. The public’s lack of comfort with scientific disagreement further compounds distrust. Rather than embracing uncertainty as a natural component of scientific progress, conflicting expert opinions can be perceived as a sign of incompetence or unreliability. This has led many to prioritize personal anecdotes and social influence over evidence-based decision-making. We as a scientific community need to “strip the veil” from science—to make it transparent, accessible, and culturally resonant. Leaders in sociology who study vaccine hesitancy and science communication argue for embracing more public-facing venues for communication, such as commentaries, op-eds, and social media. Trust must be built through engagement, not explanation alone.

Science should be made visible and engaging early in education, emphasizing science and math that people already encounter in daily life (e.g., cooking or managing a household budget) as a gateway to broader understanding. The use of stories is a powerful tool. Narratives not only level the playing field between experts and the public but also allow for emotion, values, and nuance to coexist with data. Scientists must become relatable and communicate the science in ways that resonate with people’s experiences and concerns. Trust in science must also be cultivated through local and decentralized engagement. Community health workers, religious leaders (55), and other trusted local figures can act as cultural translators or “chaperones” for scientific messages. However, these relationships should be collaborative: scientists bring technical expertise, and community leaders bring context and trust, as well as an audience and structure in which to operate. These partnerships also help to validate scientists.

Transparency in community-based research is also essential, particularly in sharing results and implications with participants and stakeholders. Chronic underfunding of the public scientific enterprise has fueled dependency on industry, contributing to public suspicion. Although partnerships with industry are important, greater transparency is needed to explain the safeguards in place to protect scientific integrity. Teaching young scientists about funding diversity and accountability should become a core component of their training. Additionally, scientists must be prepared to explain procedural deviations such as the compressed timelines during COVID-19 vaccine development in ways that are both technically accurate and publicly comprehensible. Being proactive in communication, especially before crises unfold, is a necessary evolution for building trust.

One major barrier to engaging in science communication is the time and resource burden on individual scientists. Institutions should formalize support by incorporating communication efforts into graduate education, as well as promotion and tenure frameworks, and by offering protection and mentorship for those engaging in potentially controversial public dialog. Collaboration is a way to reduce burden; group-authored commentaries, shared platforms, and partnerships with communication professionals can amplify messages while distributing workload. A culture shift is needed to recognize science communication as a scholarly and impactful contribution, not an extracurricular activity. Restoring trust in microbiology and public health requires a paradigm shift: from gatekeeping to engagement, from abstract data to shared stories, and from centralized authority to community-informed action.

Finally, transparent and relatable communication is essential for supporting policy and advocacy. Educating elected officials, many of whom lack formal scientific training, is vital. Scientists should frame their work in terms that resonate with policymakers, such as local economic or health impacts. Successful examples, such as science education sessions for legislators hosted by museums, demonstrate how such engagement can be impactful and nonpartisan. Maintaining consistency in scientific messaging across political cycles is critical to preserving public trust, and science should be presented as apolitical, with a focus on facts rather than rebutting myths directly. Leveraging local media and trusted community voices is recommended as a more effective strategy than relying solely on national platforms.

Through transparent communication, early and inclusive education, localized partnerships, and institutional reforms, we can help bridge the gap between science and society, empowering individuals to make informed decisions while strengthening the social fabric around evidence-based practice.

## KEY RECOMMENDATIONS

**Fig 3:**
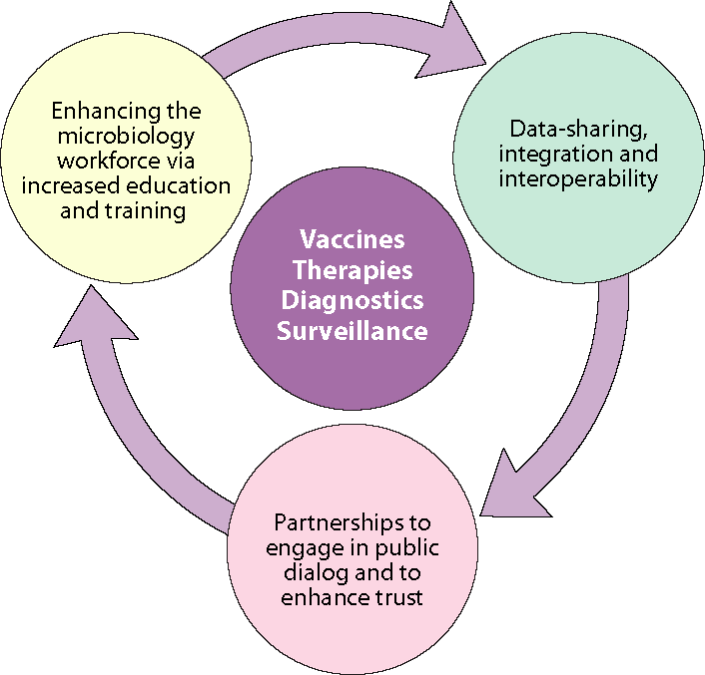
Schematic diagram showing the central focus of the ASM Health Unit as improving vaccines, therapies, diagnostics, and surveillance. Success will be greatly enhanced by the activities in the outer circle, with each influencing the other two.

To illustrate how data transformation, enhancement of the microbial workforce, and public education can influence and enhance scientific progress in the core activities of infectious disease and public health microbiology, we developed a schematic diagram showing the integration of the retreat topics (Fig. 3). Major recommendations for ASM are noted below.

### How do we support the development of the most critical interventions for microbial diseases?

In a world of growing disease prevalence, reduced investment in antimicrobials, and declining vaccine uptake, it is crucial that a unified scientific community prioritizes what is essential for public health, based on threats to humans and animals. To address the world’s deadliest infectious diseases, we need to work in tandem with global surveillance for diseases like tuberculosis, malaria, and HIV, as well as polio, measles, influenza, and emerging viruses. Some of the technologies for vaccines and monoclonal antibodies that were fast-tracked for SARS-CoV-2 could be used to tackle other persistent and emerging pathogens (2). Collaboration with both public and private sectors is needed to optimize funding and resources, particularly with significantly reduced government funding for research and surveillance. On the local level, we need to address regional needs and delivery methods, with an emphasis on overcoming vaccine hesitancy. Innovations in genomic sequencing, wastewater surveillance, and point-of-care diagnostics improve detection, and these are global, not regional, issues. There are existing programs in other organizations, such as the WHO and CDC, but these programs are either experiencing or under threat of reduced funding, and ASM could play a critical partnership role by advocating for their continued support and by providing expertise, advocacy, and strengthening international collaborations.

### How can we bridge the gap to achieve true interoperability and data sharing?

The value of coordination, collaboration, and data sharing cannot be overstated, and there are critical gaps in the uniformity of data, data management, and operational services, which are not fluid across institutions, regions, and/or countries. Managing public health emergencies effectively requires common standards for data collection, standardized data handling systems and interoperability, and robust public health IT infrastructure (4). Success will require a long-term strategy and major investments on a system-wide level. As human disease databases and information synthesis platforms have continued to become more comprehensive and integrated into health systems, there is a significant need to establish similar platforms for animal infectious diseases. Many of the same conclusions were drawn by the ASM Host-Pathogen community retreat (56). ASM could be instrumental in identifying federal, clinical, and public health partners, facilitating standardization of information exchange systems, obtaining funding, and advocating for microbiologists to serve as experts when building systems to support population and public health. Examples of successes could be shared at Microbe or at other ASM conferences and featured in publications.

### How do we build and sustain the microbiological workforce?

As we look to the future of the microbiology workforce, there is a growing demand for professionals in clinical laboratories—not only for MLS but also for individuals with doctoral training in microbiology. To meet this need, ASM has an opportunity to help reshape academic training programs to better align with evolving career landscapes, placing less emphasis on traditional academic paths. A key step in this transformation is to clearly communicate the value of diverse career opportunities across academia, industry, clinical laboratories, public health, and government. To prepare a workforce that is both adaptable to change and inspired, it will be critical to break down disciplinary silos, promote cross-training, and foster a culture that embraces technological innovation. This is a long-term goal that will require significant funding and attention to a shifting landscape, as funding is in a state of flux due to recently imposed U.S. national priorities that de-emphasize public health. ASM can further support expanded partnerships with laboratories and institutions—such as its collaboration with Weber State (https://www.weber.edu/mls/pbcmicrotech.html)—to offer accessible, low-cost training programs for early-career professionals in laboratory science.

### How can we improve communication about science in ways that bridge knowledge gaps and political divides?

We need to regain the mantle of “experts” while sharing our knowledge of the ubiquitous microbes; one way is to keep our natural curiosity as part of the conversation and to be honest about failures and unanswered questions. Recent publications have emphasized the role that the scientific and medical communities need to play in combating vaccine revisionism (1) and public health misinformation (57). ASM is well-positioned to advocate for public-private partnerships that bring together academic, foundation, government, public health, and industry partners and to innovate in funding, both for scientific research and outreach. With its effective reach to students, ASM is also poised to play a major role in developing effective communication programs and resources for those engaged in public outreach. ASM can contribute as a resource warehouse and can host training opportunities at conferences to share model programs directed at the restoration of trust in microbiological sciences.

In closing, although these recommendations cannot be accomplished solely by ASM, we strongly support ASM’s investment in its membership and leadership in these four areas, in an effort to assure a vital public health future.
